# Efficacy of thermoplastic polyurethane and gelatin blended nanofibers covered stent graft in the porcine iliac artery

**DOI:** 10.1038/s41598-022-20950-w

**Published:** 2022-10-03

**Authors:** Dae Sung Ryu, Dong-Sung Won, Ji Won Kim, Yubeen Park, Song Hee Kim, Jeon Min Kang, Chu Hui Zeng, Dohyung Lim, Hyun Choi, Jung-Hoon Park

**Affiliations:** 1 Biomedical Engineering Research Center, Asan Institute for Life Sciences, Asan Medical Center, 88 Olympic-ro 43-gil, Songpa-gu, Seoul, 05505 Republic of Korea; 2 Department of Mechanical Engineering, Sejong University, 209, Neungdong-ro, Gwangjin-gu, Seoul, 05006 Republic of Korea

**Keywords:** Preclinical research, Biomedical engineering

## Abstract

Stent-grafts composed of expanded polytetrafluoroethylene (e-PTFE), polyethylene terephthalate (PET) and polyurethane (PU) are characterized by poor endothelialization, high modulus, and low compliance, leading to thrombosis and intimal hyperplasia. A composite synthetic/natural matrix is considered a promising alternative to conventional synthetic stent-grafts. This study aimed to investigate the efficacy of thermoplastic polyurethane (TPU) and gelatin (GL) blended nanofibers (NFs) covered stent-graft in the porcine iliac artery. Twelve pigs were randomly sacrificed 7 days (n = 6) and 28 days (n = 6) after stent-graft placement. The thrombogenicity score at 28 days was significantly increased compared at 7 days (*p* < 0.001). The thickness of neointimal hyperplasia, degree of inflammatory cell infiltration, and degree of collagen deposition were significantly higher at 28 days than at 7 days (all *p* < 0.001). The TPU and GL blended NFs-covered stent-grafts successfully maintained the patency for 28 days in the porcine iliac artery. Although thrombosis with neointimal tissue were observed, no subsequent occlusion of the stent-graft was noted until the end of the study. Composite synthetic/natural matrix-covered stent-grafts may be promising for prolonging stent-graft patency.

## Introduction

Synthetic vascular grafts are widely used in surgical and/or minimally invasive interventional therapeutic procedure to treat vascular diseases, such as coarctation of the aorta, aneurysm, arteriovenous fistula, dissection, iatrogenic vascular perforation, traumatic injury, and hemodialysis access^1,2^. Commercially available synthetic stent-grafts composed of expanded polytetrafluoroethylene (e-PTFE), polyethylene terephthalate (PET) and polyurethane (PU) are characterized by poor endothelialization, high modulus, and low compliance, leading to thrombosis and intimal hyperplasia^3–5^. Therefore, rapid endothelialization, good mechanical properties, thromboresistivity and biocompatibility are key to preserving the long-term patency of stent-grafts^6,7^. A composite synthetic/natural matrix is thought to be a promising alternative to conventional synthetic stent-grafts since it has sufficient mechanical properties and good cytocompatibility^8^.

Nanofibers (NFs) coatings manufactured by electrospinning (ES) can be adequately durable if the covering layer’s appropriate polymeric composition and thickness are designed^9^. Several synthetic or natural biomaterials have been used to fabricate NF scaffolds include polylactide, poly-caprolactone, poly (glycolic acid), collagen, chitosan, and gelatin (GL)^10–14^. GL is an excellent tissue engineering material owing to its unique features, such as biodegradability, biocompatibility, ability to promote cell adhesion and proliferation, and low immunogenicity^15,16^. In addition, previous research has demonstrated the GL’s ability to improve the strength of electrospun matrices^17^. Meanwhile, Tecoflex is a family of thermoplastic polyurethane (TPU) that would be a good candidate for reinforcement. As a thermoplastic elastomer, TPU has been widely used as covering materials for breast implants, catheters, and prosthetic heart valve leaflets because of its superior mechanical properties^18^. The matrix composition of the TPU and GL can alter the properties of blended metrics and may enhance the mechanical and biological properties. The optimized TPU and GL blended matrices has high potential as materials for use in engineering of elastic tissues like stent-grafts, valves, patches for vascular and non-vascular organs^18^. The TPU and GL blended NFs covering membrane have been investigated as composite 3D matrix for stent-grafts; however, this composition is yet to be studied in vivo. Therefore, the purpose of this study was to investigate the efficacy of TPU and GL blended NFs-covered stent-graft in the porcine iliac artery.

## Materials and methods

### Stent construction

The stent (S&G biotech Co., Ltd., Yongin, Korea) was woven into a tubular configuration with single thread of 0.127-mm-thick nitinol wire. When fully expanded, the stent was 6 mm in diameter and 30 mm in length. Radiopaque markers were attached to the ends and middle of the stent to facilitate visualization under fluoroscopy. The 6-Fr delivery system had a usable length of 500 mm and was comprised of an outer braided sheath and a pusher catheter with a guiding olive tip (S&G biotech Co., Ltd.).

### TPU and GL blended NFs-covering onto the stent

**Figure 1:**
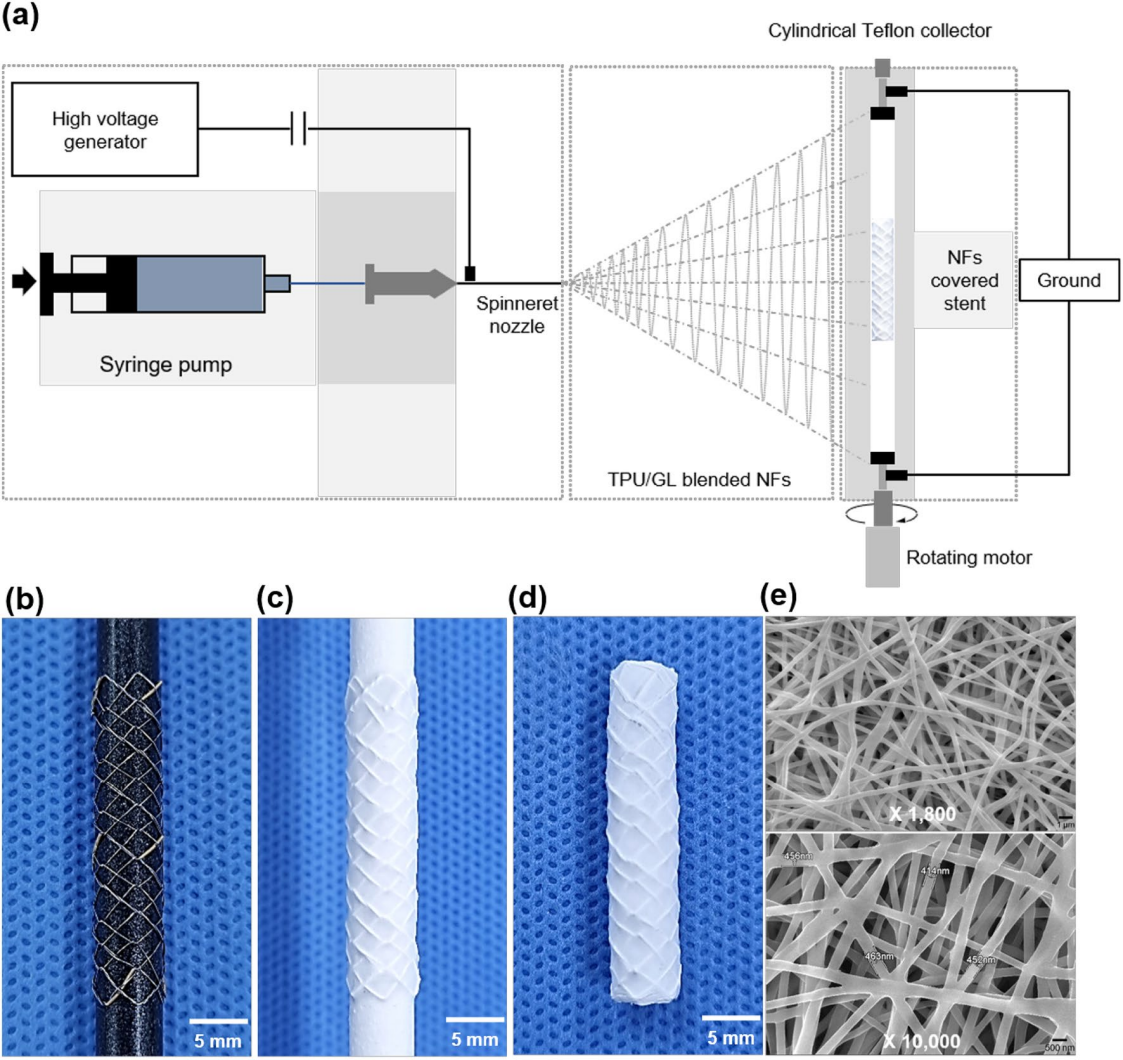
The technical steps of fabricated TPU and GL blended NFs-covered stent-graft using electrospinning. (**a**) Schematic image for fabrication of TPU and GL blended NFs-covered stent- graft. (**b**) The stent was secured on the cylindrical Teflon collector. (**c**) The stent with the cylindrical Teflon collector was fully coated. (**d**) TPU and GL blended NFs-covered stent-graft. (**e**) SEM images of TPU and GL blended NF scaffolds show randomly orientated NF structures at × 1,800 and × 10,000 magnification. Note. TPU: thermoplastic polyurethane, GL: gelatin, NF: nanofiber.

The TPU and GL blended NFs membrane was constructed using ES techniques (BFT-ES1000, S&G biotech CO., Ltd.) (Fig. 1a). The ES solutions were prepared by mixing the stock solutions of 3% TPU, (Tecoflex EG 80A; Lubrizol Corporation, OH, USA), 15% GL (G2500; Sigma Aldrich, MO, USA) and 1,1,1,3,3,3-hexafluoro-2-propanol (HFIP, 105,228, Sigma Aldrich). The matrix composition of the TPU and GL was determined with reference to previous study^16^. The stent was secured on the cylindrical Teflon collector (Fig. 1b), which constantly rotated for a uniform distribution of NFs over the entire stent surface (Fig. 1c). Then, excess NF covering layers was cut off and the covered stent-graft was vacuum dried at room temperature for 24 h. (Fig. 1d). The ES parameters were: feed rate, 1 mL/h; voltage, 15 kV; collector rotation speed, 750 rpm; distance between the collector and the spinneret, 15 cm; ES time, 30 min. Surface morphology of the NF covering layers was observed by a scanning electron microscopy (SEM; Hitachi S-2380 N, Tokyo, Japan) (Fig. 1e).

### Animal study design

A total of twelve iliac arteries of 12 Yorkshire domestic pigs weighing 35.7–37.7 kg (male; mean weight = 36.7 kg; Orient Bio, Seongnam, Korea) were used in this study and randomly sacrificed 7 days (n = 6) and 28 days (n = 6) after stent-graft placement. The TPU and GL blended NFs-covered stent-graft was placed in the left iliac artery (LIA). All pigs were housed under the same environmental circumstances (temperature of 24°C ± 2 with a 12-h day-night cycle) and were supplied with water and food ad libitum. All animals were euthanized with 75 mg/kg potassium chloride via marginal ear vein injection at 7 or 28 days after stent-graft placement.

### TPU and GL blended NFs-covered stent-graft placement

All pigs were anesthetized intramuscularly with a mixture of 50 mg/kg zolazepam and 50 mg/kg tiletamine (Zoletil 50; Virbac, Carros, France) and 10 mg/kg xylazine (Rompun; Bayer HealthCare, Leverkusen, Germany) before the experiment. An endotracheal tube was then placed, and anesthesia was maintained by inhaling of 0.5–2% isoflurane (Ifran®; Hana Pharm. Co., Seoul, Korea) with 1:1 oxygen (510 mL/kg/min). A 5-Fr micro puncture kit (Cook Medical, Bloomington, IN, USA) was used to puncture the right femoral artery under ultrasonographic guidance (iU22; PHILIPS, Amsterdam, Netherlands). Then, a 7-Fr vascular sheath (Radifocus; Terumo Co., Tokyo, Japan) was replaced under fluoroscopic guidance (FORTE; DK Medical Systems, Pyeongtaek, Korea). Intra-arterial heparin (100 U/kg; JW Pharm Co., Seoul, Korea) was administered directly into the sheath. Pre-procedural angiography was performed to confirm location of the iliac artery, and a 0.018-inch guidewire (Radifocus M; Terumo Co.) was inserted through the sheath and advanced to the femoral artery. The stent delivery system was inserted over the guidewire into the LIA and the stent was placed. Post-procedural angiography was performed to confirm the patency and location of the placed stent-graft. A vascular closure device (Angio-Seal, Terumo Co.) was used followed by manual compressed for 10 min. Antibiotics (Gentamicin, 7 mg/kg; Shin Poong Pharm Ltd., Seoul, Korea) and analgesia (Keromin, ketorolac 1 mg/kg; Hana Pharm Ltd., Seoul, Korea) were routinely administered for 3 days after the procedures.

### Follow-up angiography

Follow-up angiography via the right carotid artery was performed to evaluate stent patency and stent-related complications at 7 and 28 days after the procedure, respectively. The luminal diameters were measured and compared at the middle portion of the stented iliac arteries using RadiAnt DICOM viewer (version 2020.2; Medixant, Poznan, Poland).

### Gross and histological examinations

Surgical exploration of the abdominal aortic bifurcation and both iliac arteries was performed for gross examination of possible arterial injury and thrombosis following stent placement. Approximately 100 ml of heparin-treated (100 U/kg, JW Pharm Co., Seoul, Korea) saline was irrigated into the arteries. And, the stented LIA and normal right iliac arteries were extracted for evaluated histological examinations. The stent-grafts gently removed from the stented LIA for apparent surface thrombus. The degree of thrombosis was determined using a thrombogenicity score into 1 (mild), thrombus nonexistent or minimal; 2 (mild-to-moderate), thrombus minimal, observed to be covering 1–25% of material surface; 3 (moderate), thrombus moderate, observed to be covering 26–50% of material surface; 4 (moderate-to-severe), thrombus severe, observed to be covering 51–75% of material surface; and 5 (severe), thrombus extensive, covers 76–100% of material surface^19^. The samples perfused for 48 h using 4% formalin for fixation. The samples were axially sectioned at the middle portion of the stented LIA. Tissue samples were embedded in paraffin, cut into 5 μm-thick slices and stained with Hematoxylin and Eosin (H&E) and Masson’s trichrome (MT). Histologic evaluation using H&E included determining the thickness of neointimal hyperplasia and degree of inflammatory cell infiltration. The degree of collagen deposition was determined on MT-stained sections. The thickness of neointimal hyperplasia and the degree of inflammatory cell infiltration were calculated as the average of eight values around the circumference. The degree of inflammatory cell infiltration and degree of collagen deposition were subjectively classified into 1, mild; 2, mild to moderate; 3, moderate; 4, moderate to severe; and 5, severe according to the distribution of the inflammatory cells and collagen^20^. All scan of staining samples was performed using a digital slide scanner (Pannoramic 250 FLASH III, 3DHISTECH Ltd., Budapest, Hungary). Measurements were obtained with a digital microscope viewer (CaseViewer, 3DHISTECH Ltd.). Analyses of the histological findings were based on the consensus of three observers who were blinded to the experimental groups.

### Statistical analysis

Data were expressed as mean ± standard deviation (SD). Differences between the groups were analyzed using two-sample t-test and Mann–Whitney U test, as appropriate. A *p* value < 0.05 was considered statistically significant. Statistical analyses were performed using the SPSS software (version 27; IBM, Chicago, USA).

### Approval for animal experiments

This study was approved by the Institutional Animal Care and Use Committee (IACUC approval number 2021–13-240) and conformed to US National Institutes of Health guidelines for humane handling of laboratory animals. The study was carried out in compliance with the ARRIVE guidelines.

## Results

### Procedural outcomes and angiography findings

**Figure 2:**
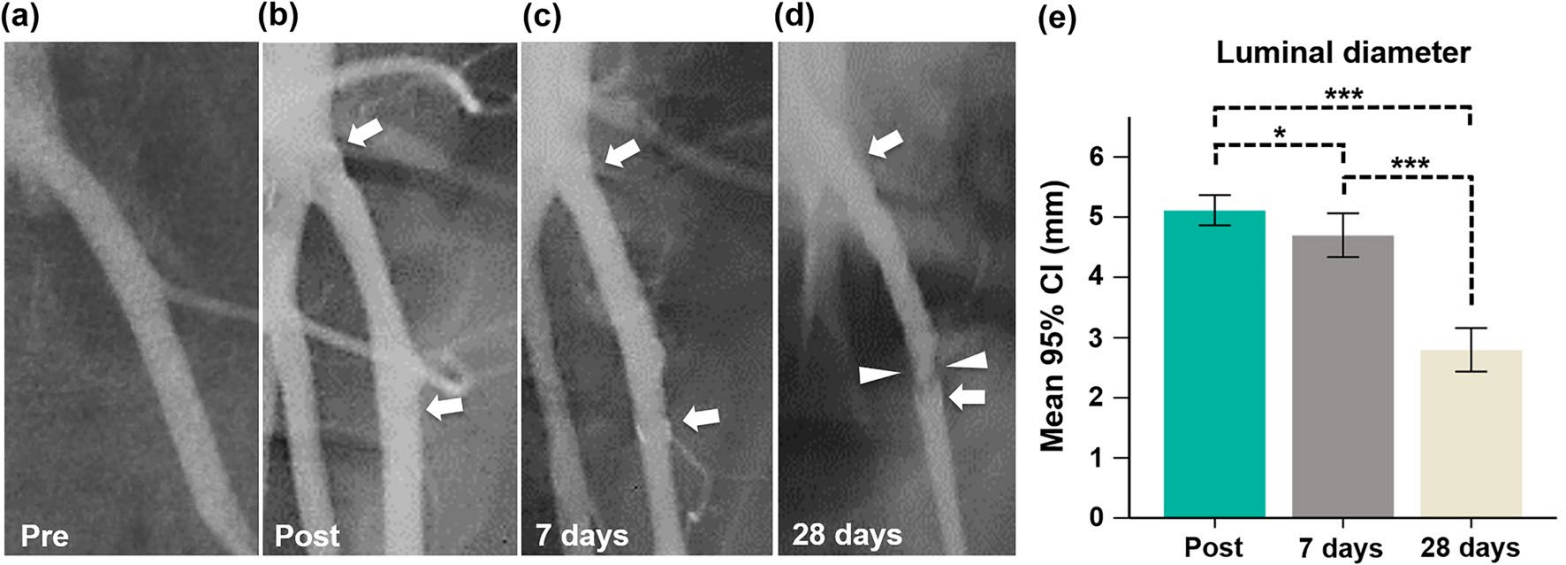
Representative follow-up angiographies with the mean luminal diameters in the stented LIAs. (**a**) Pre-angiography shows the LIA. (**b**) Post-angiography shows the fully expanded TPU and GL blended NFs-covered stent-graft (arrows). (**c**) Follow-up angiography at 7 days shows good patency of the stent-graft (arrows). (**d**) Follow-up angiography at 28 days shows an irregular luminal narrowing (arrowheads) at the distal portion of the TPU and GL blended NFs-covered stent-graft (arrows). (**e**) The graph shows changes in the luminal diameters in the stented LIAs. Note. CI, confidence interval; Note. TPU: thermoplastic polyurethane, GL: gelatin, NF: nanofiber; *p < 0.05, ***p < 0.001.

TPU and GL blended NFs-covered stent-grafts were successfully placed in all pigs without procedure-related complications. All pigs survived until the end of the study without further complications. The angiography findings are presented in Fig. 2. The mean luminal diameter gradually decreased over time. The mean (± SD) luminal diameters at 28 days follow-up angiography (2.8 ± 0.34 mm) were significantly lower than those at 7 days follow-up (4.7 ± 0.34 mm, *p* < 0.001) and post-procedure (5.1 ± 0.24 mm, *p* < 0.001) angiographies. The luminal diameter at 7 days follow-up angiography was not significantly different the post-procedure angiography (*p* > 0.05). Follow-up angiographies showed good patency of the stented LIAs without stent-related complications such as endoleak, migration, collapse and occlusion. However, irregular luminal narrowing caused by thrombosis was observed 7 days (1 of 6, 16.7%) and 28 days (5 of 6, 83.3%) after stent placement.

### Gross and histological findings

**Figure 3:**
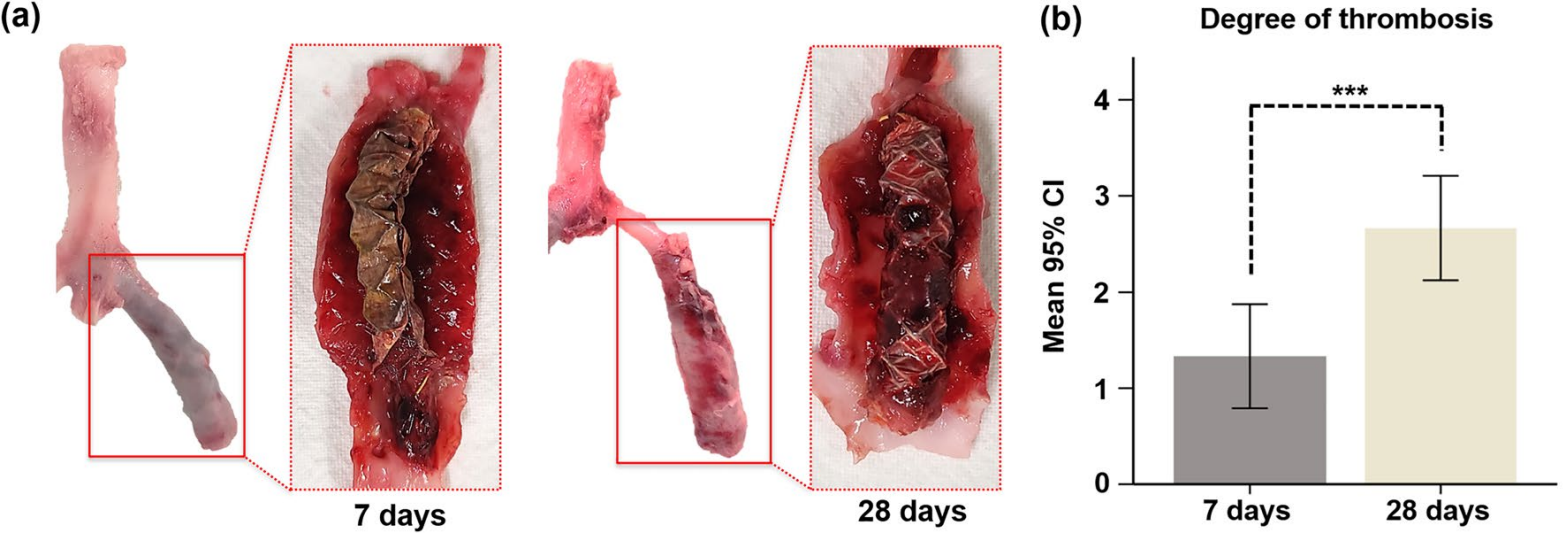
Representative images of gross specimens. (**a**) Image showing TPU and GL blended NFs-covered stent-graft with thrombosis in LIA after sacrifice for extraction at 7 days and 28 days. (**b**) The mean (± SD) degree of the thrombosis at 28 days compared with 7 days. CI, confidence interval; ***p < 0.001.

All TPU and GL blended NFs-covered stent-grafts with iliac arteries were successfully extracted. In-stent thrombosis was observed on the inner and outer walls of the stent-graft in all specimens on gross examination (Fig. 3a). The mean (± SD) thrombogenicity score was significantly increased at 28 days than at 7 days (2.67 ± 0.51 vs. 1.33 ± 0.51, *p* < 0.001) after stent placement (Fig. 3b).

**Figure 4:**
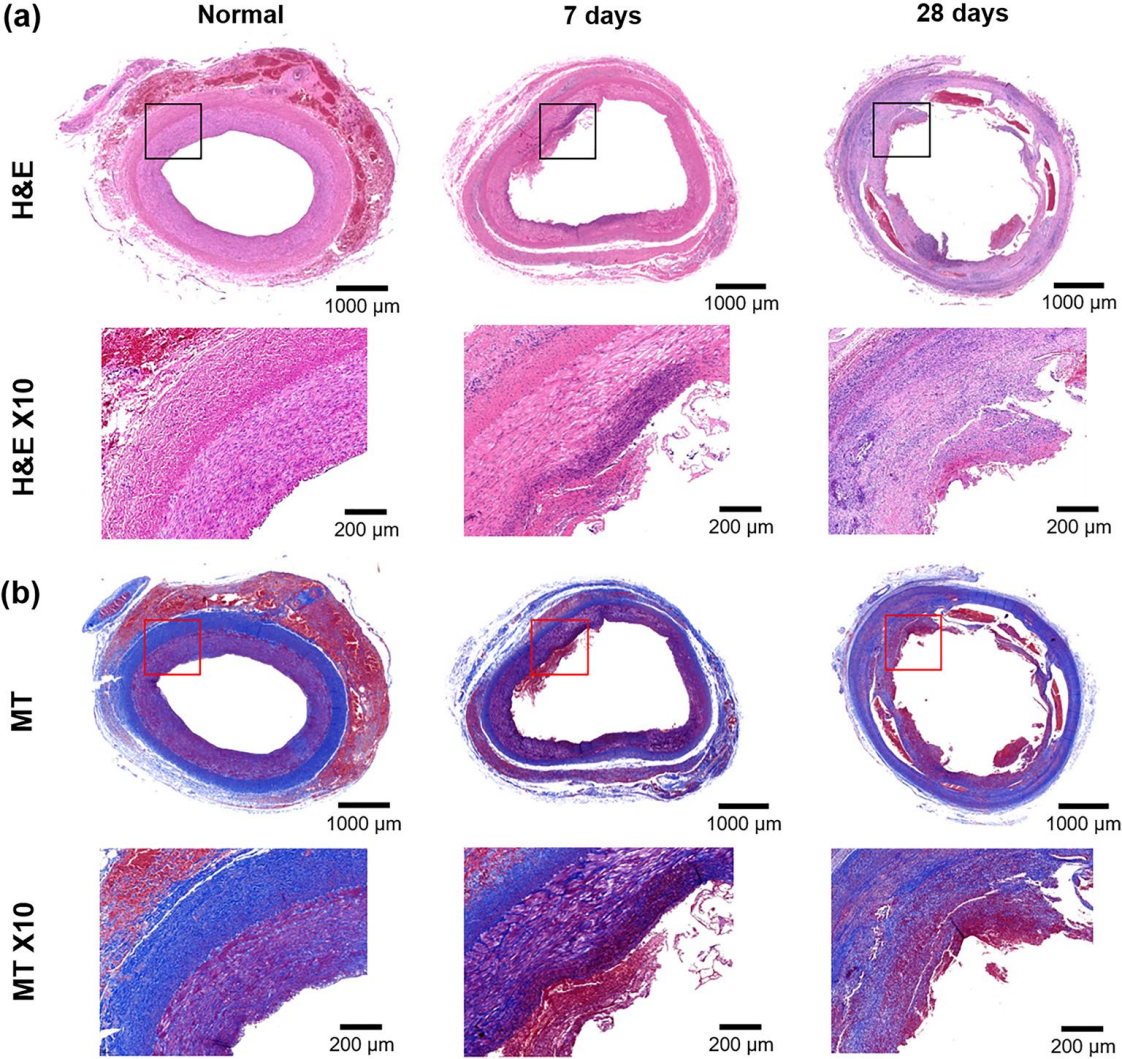
Representative images showing serial histological changes in normal iliac artery, 7 and 28 days. (**a**) Haematoxylin and eosin staining (H&E) with × 1 and × 10 magnifications. × 10 images indicate locations for the higher magnification (black boxes). (**b**) Masson trichrome staining (MT) with × 1 and × 10 magnifications. × 10 images indicate locations for the higher magnification (red boxes).

**Figure 5:**
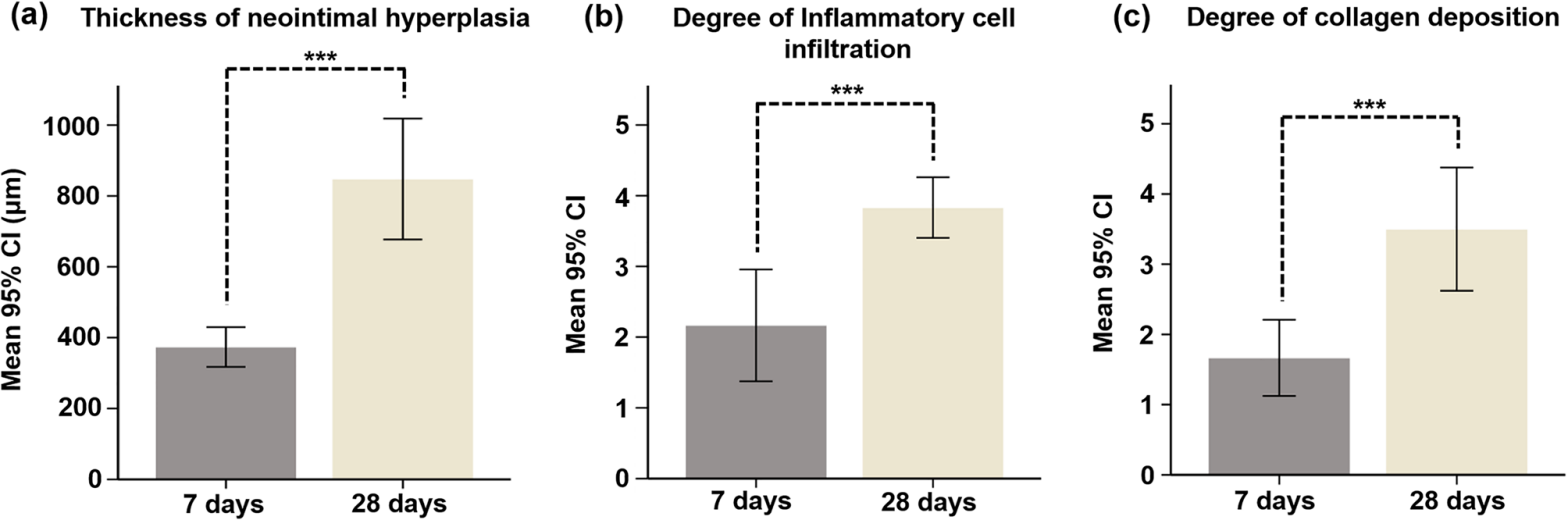
Histological findings. (**a**–**c**) Histological results of the LIA on days 7 and 28. CI, confidence interval; ***p < 0.001.

Histological findings are presented in Figs. 4 and 5. The mean (± SD) thickness of neointimal hyperplasia was significantly higher at 28 days than at 7 days (847.8 ± 162.6 μm vs. 373.7 ± 53.25 μm, *p* < 0.001) (Fig. 5a). Furthermore, the degree of inflammatory cell infiltration (3.66 ± 0.51 vs. 1.83 ± 0.40, *p* < 0.001) (Fig. 5b) and degree of collagen deposition (3.50 ± 0.83 vs. 1.33 ± 0.51, *p* < 0.001) (Fig. 5c) were significantly higher at 28 days than at 7 days.

## Discussion

The present study results demonstrated that the patency of the TPU and GL blended NFs-covered stent-graft was well preserved for 28 days; however, thrombus with mild neointimal hyperplasia gradually increased over time. Follow-up angiography revealed irregular luminal narrowing into the stent-graft and the luminal diameter of the stented iliac artery was significantly lower at 28 days than at 7 days of follow-up angiography. However, stent occlusion was not observed in any of the pigs. Consistent with the gross findings, histological results demonstrated significantly increased neointimal hyperplasia with prominent inflammatory cells and collagen deposition in the 28 days group, which correlated with the angiographic findings.

Commercially available and conventional synthetic stent-grafts covered by e-PTFE or PET have been commonly used for large diameter arteries (> 6 mm) and applied for treatment of endovascular diseases^21^. However, their bulky design can make deliverability to tortuous. Furthermore, stent-in-restenosis was frequently occurred in case of relatively small diameter arteries (< 6 mm) caused by high possibility of calcified or thrombogenicity of the grafts^22^. The incidence of restenosis rate of stent-graft covered with e-PTFE was 12% with thrombosis within a month^23^. Wong et al. also reported that the luminal diameter of e-PTFE covered stent-grafts in the porcine carotid arteries was reduced to 67.2% caused by neointimal hyperplasia with inflammatory reaction^24^. Other complications related with conventional stent-graft include thrombosis, restenosis and delayed endothelialization^25^. Therefore, developing new synthetic stent-grafts with improved stent patency and hemocompatibility is becoming increasingly important^26^. Our preliminary study revealed that the TPU and GL blended NFs-covered stent-graft with the optimized mechanical and biological properties was successfully maintained the patency without significant stent-in-restenosis for 28 days. However, long-term inflammatory response with possible complications should be validated for clinical application.

ES has been widely used to fabricate NF membranes because of it high porosity, large surface area, nanofibrous structure of electrospun scaffolds, and its mimicking of the physical nano features of the native extracellular matrix^27^. Furthermore, the simplicity of the ES technique for fabricating a tubular scaffold makes it more popular for vascular tissue regeneration applications^28^. TPU is commonly used in medical tubing because of its high hemocompatibility. In addition, its non-biodegradability, which may also contribute to blood vessel replacement is required^29^. Radeleff et al. reported the TPU covered stent-grafts compared over self-expandable metallic bare stents in a 28 days porcine model. However, TPU stent-grafts failed to improve patency due to excessive neointimal hyperplasia and subsequent occlusion^15^. Despite the relatively good cell viability of TPU, its poor adhesion and proliferation of endothelial cells affects the antithrombogenic properties of vascular structures^30,31^. In this case, finding an appropriate material to pair with TPU and convert it into a synthetic stent-graft may be a solution to improve its efficacy for endovascular treatment. Recently, in the context of employing at composite mesh as a vascular graft, TPU and GL were mixed to obtain a composite natural/synthetic polymer matrix^16^. In addition to promoting adhesion and proliferation of endotheliocytes, GL also plays a role in strengthening the electrospun matrices^17^. Our study successfully fabricated TPU-and GL-blended NFs-covered stent grafts using ES and showed short term patency without stent-related complications such as migration, endoleak and collapse.

This study had several limitations. First, we did not evaluate the biocompatibility and mechanical characterization of TPU and GL blended NF scaffolds in an in vitro. Second, the follow-up duration might be too short to evaluate a meaningful neointima proliferation and thrombosis formation. Further studies with longer follow-up periods are required to confirm the efficacy and safety of TPU and GL blended NFs-covered stent-graft. Third, there was no comparison with commercially available stent-grafts in our study. Fourth, the experiment was conducted in normal iliac arteries. Our findings may not reflect all pathological mechanisms that occur in humans following stent placement. Finally, the number of animals was too small to perform a robust statistical analysis. Although additional studies are required to further validate our current data, our findings provide the basic concept of TPU and GL blended NFs-covered stent-graft in the porcine iliac artery.

The TPU and GL blended NFs-covered stent-grafts successfully maintained patency for 28 days without stent graft-related complications in a porcine iliac arterial model. Although thrombosis with neointimal tissue were observed, no subsequent occlusion of the stent-graft was noted until the end of the study. Although further preclinical studies are needed to investigate its efficacy and safety, a composite synthetic/natural matrix-covered stent-graft may be promising for prolonging stent-graft patency.

## Data Availability

The datasets generated and/or analyzed during the current study are available from the corresponding author on reasonable request.
